# The Feeding Patterns as a Dominant Factor in the Occurrence of Stunting in Toddlers in Ponorogo

**DOI:** 10.1155/jnme/7589972

**Published:** 2026-03-01

**Authors:** Kurnia Dwi Artanti, Dyah Silviananda Widhiastuti, Devina Dwi Kurnia, Arina Mufida Ersanti, Taufiq Hidayat

**Affiliations:** ^1^ Division of Epidemiology, Department of Epidemiology, Biostatistics, Population and Health Promotion, Faculty of Public Health, Universitas Airlangga, Surabaya, Indonesia, unair.ac.id; ^2^ Epidemiology Integrated Intervention and Technology Collaboration (Epitech) Research Group, Faculty of Public Health, Universitas Airlangga, Surabaya, Indonesia, unair.ac.id; ^3^ Undergraduate Public Health Program, Faculty of Public Health, Universitas Airlangga, Surabaya, Indonesia, unair.ac.id; ^4^ Master of Public Health Program, Faculty of Public Health, Universitas Airlangga, Surabaya, Indonesia, unair.ac.id; ^5^ Department of Paediatrics, Kulliyyah of Medicine, International Islamic University Malaysia, Kuantan, Malaysia, iium.edu.my

## Abstract

**Introduction:**

Stunting in Indonesia, caused by nutritional deficiencies from pregnancy until the age of two, remains above the WHO target despite a decrease in prevalence from 37.2% in 2017 to 21.6% in 2022. The detrimental impact of stunting on child development and the high rates in Indonesia underscore the importance of improving maternal and child nutrition to reduce this prevalence.

**Objective:**

The objective of this study was to analyze the effect of feeding patterns on the incidence of stunting in toddlers in the Ponorogo District.

**Methods:**

This research is an analytical observational study with a case‐control study design. The sample size was calculated using the case‐control sample size formula with a 1:1 ratio, resulting in 44 cases and 44 control samples. The case samples consist of children aged 1–5 years who experience stunting in the North Ponorogo Community Health Center area. The control samples consist of children aged 1–5 years who do not experience stunting in the same area. The sampling method used is simple random sampling. Risk factor analysis uses the odds ratio (OR) value and a 95% confidence interval (CI).

**Results:**

There is an association between feeding patterns and the incidence of stunting, with an OR value of 14.54 (95% CI = 3.11 < OR < 67.86). There is no association between immunization completeness status and the incidence of stunting, with an OR value of 0.42 (95% CI = 0.14 < OR < 1.25).

**Conclusions:**

Based on the conducted research, it can be concluded that there is a significant relationship between feeding patterns and the incidence of stunting in the North Ponorogo Community Health Center area. Inadequate feeding patterns significantly increase the risk of stunting.

## 1. Introduction

Stunting is a major health issue in Indonesia, primarily due to nutritional deficiencies from pregnancy until a child reaches 2 years of age. Although its prevalence decreased from 37.2% in 2017 to 19.8% in 2024, this figure reached the WHO target of under 20%, making it an urgent issue to address [1]. According to UNICEF, WHO, and the World Bank, Indonesia has the second‐highest stunting prevalence in Southeast Asia after Timor‐Leste, with a stunting rate among children under five reaching 31.8% in 2020 [2]. This prevalence gradually decreased to 21.6% in 2022, yet ongoing efforts are needed to achieve the target of eliminating malnutrition by 2030 [3]. At the local level, East Java Province has become a priority area in stunting management, particularly in Ponorogo Regency, where stunting prevalence dropped from 21% in 2021 to 14.2% in 2022. Despite this improvement, East Java’s stunting prevalence still falls short of the targets set in the 2022 Strategic Plan, indicating that this region requires further intervention [4].

Key factors contributing to stunting include maternal health and nutrition, inadequate feeding practices, and infections in toddlers. Feeding patterns play a crucial role in the study of stunting, particularly because they serve as fundamental determinants of nutritional outcomes in young children. A study in Indonesia found a significant relationship between poor feeding patterns and the incidence of stunting, indicating that 35% of surveyed toddlers were categorized as stunted due to inappropriate feeding practices [5]. Furthermore, parenting styles, including feeding habits and frequency, significantly correlate with nutritional outcomes, establishing a direct connection between parental feeding practices and child growth [6, 7]. Inadequate dietary diversity has also been identified as a salient factor associated with stunting among children, emphasizing the necessity for varied nutrient‐rich diets [8].

Vaccination can mitigate the risk factors associated with stunting, particularly infectious diseases that lead to poor nutritional status and growth faltering. For instance, Siddiqui et al. demonstrate that vaccination significantly influences child health, reducing stunting risks among vaccinated populations, thereby enhancing weight and height growth in children [9]. Diseases such as *Shigella* and *Campylobacter* are associated with severe diarrhea that can impair nutrient absorption and growth. Anderson et al. highlighted that *Shigella-*attributable diarrhea is a significant contributor to childhood stunting [10]. If a vaccine against *Shigella* is introduced, it could potentially avert both mortality and the stunting associated with repeated infections. In addition, a study found that *Campylobacter* vaccination can reduce severe diarrheal disease and associated growth stunting, showing promise for long‐term health benefits for child nutrition [11]. A community‐based study in Burkina Faso found that improved immunization coverage accounted for 23% of advancements in height‐for‐age among children, linking better vaccination uptake with reduced stunting rates [12].

Based on this background, further research is needed to analyze the impact of feeding practices and immunization completeness on stunting incidence in toddlers, especially in the North Ponorogo Community Health Center area, which continues to face challenges in reducing stunting prevalence.

## 2. Methods

This study employs an analytical observational method with a case‐control approach, comparing case and control groups based on exposure status. In this research, the aim is to analyze the influence of feeding patterns and immunization completeness on the incidence of stunting in toddlers in Ponorogo Regency.

The sample size was calculated using the case‐control study (use sample size determination in health studies by Lemeshow):

(1) n=Z12−α/22P12−P+Z1−βP111−P+P12−P2P12−P2.

The ratio of the number of case and control samples is 1:1, so the number of case samples is 44, and the number of control samples is 44. Thus, the total number of case and control samples is 88 toddlers.

The case samples consist of toddlers aged 1–5 years who experience stunting in the working area of the North Ponorogo Community Health Center, while the control samples consist of toddlers aged 1–5 years who do not experience stunting in the same area. The sampling method used is simple random sampling. Risk factor analysis uses the odds ratio (OR) and a 95% confidence interval (CI).

Feeding patterns are defined as the nutritional intake of foods consumed by toddlers according to their age. Measurements were made using the Child Feeding Questionnaire (CFQ), which was further categorized into 2 feeding patterns: Inappropriate (< 55%) and appropriate (≥ 55%). The CFQ was used to evaluate feeding‐related behaviors and practices, not dietary intake. Research by Bodnaruc et al. highlights the established reliability and validity of the CFQ, emphasizing its role in understanding parental beliefs and attitudes toward feeding [13]. Operationally, the feeding pattern in this study is defined as a composite measure based on three indicators: type of food, amount of food, and meal schedule, which together reflect the adequacy of toddler feeding practices.

Data collection was conducted using both primary and secondary data. Primary data were obtained directly through interviews and questionnaires given to study respondents, covering feeding patterns that include meeting nutritional intake requirements for toddlers according to their age. Secondary data included data on stunted toddlers gathered from toddler weighing records; toddler characteristics such as age (calculated from birth), gender, birth weight (toddler’s weight at birth), and birth length (toddler’s length at birth), taken from the toddler cohort book; and immunization completeness status, i.e., types of immunizations received by toddlers according to their age. This research was approved by the Ethics Committee of the Faculty of Dental Medicine Universitas Airlangga with letter number: 126/HRECC.FODM/III/2021. The analysis used in this study includes descriptive analysis and bivariate analyses.

## 3. Result

Based on Table 1, most toddlers were in the 37–60 months age group (25%), with a nearly equal proportion of boys (22.7%) and girls (27.3%). The majority of stunted toddlers had a history of low birth weight (48.9%) and low birth length (43.2%), indicating that birth conditions play an important role in a child’s nutritional status. In addition, toddlers with normal nutritional status were more likely to have adequate feeding patterns, including type of food (35.2%), amount of food (30.7%), and meal schedule (31.8%), compared to stunted toddlers. These findings suggest that inadequate feeding patterns are associated with a higher incidence of stunting, emphasizing the importance of providing a balanced, sufficient, and regular diet to prevent stunting among toddlers.

**TABLE 1:** Toddler characteristics in stunting incidence in the North Ponorogo Community Health Center working area, 2021.

Variable	Status		Total
	Normal	Stunting	
*Toddler age*			
a. Baby (0–12 months)	9 (10.2%)	11 (12.5%)	20 (22.7%)
b. Under three years (13–36 months)	17 (19.3%)	11 (12.5%)	28 (31.8%)
c. Under five years (37–60 months)	18 (20.5%)	22 (25%)	40 (45.5%)

*Gender of the toddler*			
a. Boy	23 (26.1%)	20 (22.7%)	43 (48.9%)
b. Girl	21 (23.9%)	24 (27.3%)	45 (51.1%)

*Birth weight history of the toddler*			
a. Low birth weight	43 (48.9%)	43 (48.9%)	86 (97.7%)
b. Normal birth weight	1 (1.1%)	1 (1.1%)	2 (2.3%)

*Birth length history of the toddler*			
a. Low birth length	42 (47.7%	38 (43.2%)	80 (90.9%)
b. Normal birth length	2 (2.3%)	6 (6.8%)	8 (9.1%)

*Feeding pattern*			
Type of food			
a. Adequate	31 (35.2%)	29 (33%)	16 (68.2%)
b. Inadequate	13 (14.8%)	15 (17%)	28 (31.8%)
Amount of food			
a. Adequate	27 (30.7%)	23 (26.1%)	50 (56.8%)
b. Inadequate	17 (19.3%)	21 (23.9%)	38 (43.2%)
Meals schedule			
a. Adequate	28 (31.8%)	22 (25%)	50 (56.8%)
b. Inadequate	16 (18.2%)	22 (25%)	38 (43.2%)

In Table 2, the cross‐tabulation between feeding patterns and stunting incidence shows that among toddlers experiencing stunting, 18 respondents (40.9%) had inadequate feeding patterns, while 26 respondents (59.1%) had adequate feeding patterns.

**TABLE 2:** Cross‐tabulation of feeding patterns and immunization completeness status with stunting incidence in toddlers in the North Ponorogo Community Health Center working area, 2021.

Variable	Stunting Incidence				*p* value	OR	95% CI
	Yes		No				
	*n*	(%)	*n*	(%)			
*Feeding patterns*							
Inadequate	18	40.9	2	4.5	< 0.001	14.5	3.11 < OR < 67.86
Adequate	26	59.1	42	95.5			

*Immunization completeness status*							
Incomplete	6	13.6	12	27.3	0.19	0.4	0.14 < OR < 1.25
Complete	38	86.4	32	72.7			

In contrast, among toddlers not experiencing stunting, 2 respondents (4.5%) had inadequate feeding patterns, while 42 respondents (95.5%) had adequate feeding patterns. Bivariate analysis results show a *p* value of < 0.001 (0.00 < 0.05), indicating a significant relationship between feeding patterns and stunting incidence in toddlers. Furthermore, the OR analysis indicates a value of 14.54 (95% CI = 3.11 < OR < 67.86). This OR value is significant. This means that there is an effect of feeding patterns on stunting incidence in toddlers. Toddlers with inadequate feeding patterns have a 14.54 times higher risk of experiencing stunting compared to those with adequate feeding patterns. The results indicate that feeding patterns are a risk factor for stunting among toddlers in the North Ponorogo Community Health Center working area.

The cross‐tabulation between immunization completeness status and stunting incidence shows that among toddlers experiencing stunting, 6 respondents (13.6%) had incomplete immunization, while 38 respondents (86.4%) had complete immunization status. Among toddlers not experiencing stunting, 12 respondents (27.3%) had incomplete immunization status, while 32 respondents (72.7%) had complete immunization status. Bivariate analysis results show a *p* value of 0.19 (0.19 > 0.05), indicating no significant relationship between immunization completeness status and stunting incidence in toddlers. In addition, the OR analysis yields a value of 0.42 (95% CI = 0.14 < OR < 1.25), which is not significant.

## 4. Discussion

Feeding patterns play a crucial role in meeting nutritional needs, where the body’s nutrient intake depends heavily on the quantity and quality of food and drink consumed. This directly impacts an individual’s health. Balanced nutrition in feeding patterns can improve health and prevent various chronic diseases, particularly those related to nutrition [1].

Research indicates a relationship between feeding patterns and stunting incidence in toddlers, meaning toddlers with improper feeding patterns are 14.54 times more likely to experience stunting than those with proper feeding. This aligns with Nurdiana’s findings that improper feeding patterns significantly increase the risk of stunting, with affected toddlers facing a 5.6 times greater risk than those with proper feeding patterns [14].

Some research in Indonesia found that feeding practices, such as breastfeeding and complementary feeding, healthy eating habits, nutritious food choices, and portion management, play a role in improving a child’s nutritional status [15, 16]. This is the same as the research in Mexico, which illustrated that feeding practices, specifically breastfeeding, are closely associated with stunting rates, showcasing the potential impact of dietary interventions in reducing undernutrition [17]. Moreover, Nuraini et al. highlighted that inadequate nutrition, particularly during the transition from breastfeeding to complementary feeding, is a significant stunting factor. Their multivariate analysis revealed that inadequate nutrition notably impacts stunting, with an OR of 3.429, indicating that children with poor feeding practices are over three times more likely to experience growth delays [18].

Research underscores that minimum dietary diversity significantly impacts stunting rates. A study indicated that children with inadequate dietary diversity were at an increased risk of stunting, reinforcing the need for varied food intake in early childhood [8]. Furthermore, other studies emphasize the criticality of timing for complementary feeding and the necessity for exclusive breastfeeding for the first 6 months to optimize child nutrition and minimize stunting [19, 20].

Feeding patterns and stunting are also profoundly influenced by socioeconomic factors and maternal education levels. Research illustrates that mothers with higher educational attainment are more likely to adopt beneficial feeding practices, which correlate with lower stunting rates [21]. Conversely, low socioeconomic status has been associated with inadequate dietary intake, reinforcing the link between economic disparities and child malnutrition [22, 23].

Vaccination can mitigate the risk factors associated with stunting, particularly infectious diseases that lead to poor nutritional status and growth faltering. Research conducted in various countries underscores the importance of comprehensive vaccination programs in improving health outcomes. For example, in Nepal, despite facing socioeconomic challenges, the introduction of various health initiatives, including immunization, correlated with significant reductions in stunting prevalence, from 66% in 1996 to 36% in 2016 [24]. A community‐based study in Burkina Faso found that improved immunization coverage accounted for 23% of advancements in height‐for‐age among children, linking better vaccination uptake with reduced stunting rates [12].

Other studies emphasized the broader implications of immunization on child health. Vaccination programs that successfully cover populations can reduce the incidence of vaccine‐preventable diseases, which may help decrease the burden of malnutrition associated with disease‐related growth stunting [25]. Notably, proper maternal and child health services, including immunizations, were shown to correlate positively with stunting outcomes [26]. The potential economic benefits of vaccines targeting infections linked to stunting are significant. For example, Puett et al. highlight that vaccines leading to reductions in *Shigella*‐related stunting may prove more cost‐effective when accounting for future productivity benefits resulting from improved child growth outcomes [27]. Similarly, health economic modeling indicates that proper immunization strategies yield substantial long‐term benefits concerning economic burden and health enhancement in vulnerable populations [28]. The limitation of this research was that methods such as 24‐h dietary recall, FFQ, or weighed food records would provide more precise intake measurements and should be considered in future studies.

## 5. Conclusions

Research concludes that those feeding patterns were a dominant factor influencing the occurrence of stunting among toddlers. Interventions targeting improvements in feeding practices, dietary diversity, and maternal education are imperative for addressing this critical public health issue. As stunting continues to pose significant health risks in various populations, comprehensive strategies that incorporate educational and nutritional interventions are essential for effective prevention and management.

## Author Contributions

Kurnia Dwi Artanti and Dyah Silviananda Widhiastuti designed the study and wrote the protocol, and all authors performed the study. Kurnia Dwi Artanti supervised all the steps in the review process, and all authors interpreted the findings. Kurnia Dwi Artanti, Dyah Silviananda Widhiastuti, Devina Dwi Kurnia, Arina Mufida Ersanti, and Taufiq Hidayat drafted the manuscript. Kurnia Dwi Artanti supervised the writing, and Taufiq Hidayat provided feedback.

## Funding

No funding was obtained for this study.

## Disclosure

All authors read and approved the final manuscript.

## Ethics Statement

The study is exempt from review by Universitas Airlangga Faculty of Dental Medicine Health Research Ethical Clearence Commission with no 126/HRECC.FODM/III/2021, and waived the need for informed consent.

## Consent

All participants gave informed written consent to participate in the study.

## Conflicts of Interest

The authors declare no conflicts of interest.

## Data Availability

Data will be available on demand from the corresponding author.
